# Association of personal and systemic factors on intrapartum risk perception and obstetric intervention rates: a cross-sectional study

**DOI:** 10.1186/s12884-024-06338-w

**Published:** 2024-02-22

**Authors:** Nina H Peterwerth, Margareta Halek, Rainhild Schäfers

**Affiliations:** 1 Department of Applied Health Sciences - Midwifery, University of Applied Sciences-Hochschule für Gesundheit, Gesundheitscampus 6-8, 44801 Bochum, Germany; 2 School of Nursing Science, Faculty of Health, Department für Pflegewissenschaft, Fakultät für Gesundheit, Witten/Herdecke University, Alfred-Herrhausen-Straße 50, 58455 Witten, Germany; 3 Institute of Midwifery Science, Faculty of Medicine, University of Münster, Malmedyweg 17-19, 48149 Münster, Germany

**Keywords:** Decision making, Midwifery, Healthcare professionals, Risk, Obstetricians, Childbirth

## Abstract

**Background:**

Risk perception is fundamental to decision-making; therefore its exploration is essential to gaining a comprehensive understanding of the decision-making process for peripartum interventions. The aim of this study was to investigate associations between personal and systemic factors of the work setting and the risk perception of obstetric healthcare professionals, and in turn how this might influence decisions regarding obstetric interventions.

**Methods:**

Case vignettes were used to measure risk perception. A quantitative cross-sectional online survey was performed within an exploratory sequential mixed-methods design, and an intervention readiness score created. Associations were calculated using location and dispersion measures, t-tests and correlations in addition to multiple linear regression.

**Results:**

Risk perception, as measured by the risk assessment score, was significantly lower (average 0.8 points) for midwives than for obstetricians (95%-CI [-0.673; -0.317], *p* < .001). Statistically significant correlations were found for: *years of experience* and *annual number of births in the current workplace*, but this was not clinically relevant; *hours worked*, with the groups of participants working ≥ 30,5 h showing a statistically significant higher risk perception than participants working 20,5–30 h (*p* = .005); and *level of care of the current workplace*, with the groups of participants working in a birth clinic (Level IV) showing a statistically significant lower risk perception than participants working in Level I hospital (highly specialised obstetric and neonatal care; *p* = .016). The option of *midwife-led birthing care* showed no correlation with risk perception. The survey identified that risk perception, occupation, years in the profession and number of hours worked (i.e. full or part time) represent significant influences on obstetric healthcare professionals’ willingness to intervene.

**Conclusions:**

The results of the survey give rise to the hypothesis that the personal and systemic factors of professional qualification, occupation, number of hours worked and level of acuity of the workplace are related to the risk perception of obstetric healthcare professionals. In turn, risk perception itself made a significant contribution to explaining differences in willingness to intervene, suggesting that it influences obstetricians’ and midwives’ decision-making. Overall, however, the correlations were weak and should be interpreted cautiously. The significant variations in the use of interventions must be addressed in order to provide the highest quality and best possible care for childbearing women and their families. To this end, developing strategies to improve interdisciplinary relationships and collaboration is of great importance.

**Trial registration:**

German Clinical Trials Register DRKS00017172 (18.06.2019).

**Supplementary Information:**

The online version contains supplementary material available at 10.1186/s12884-024-06338-w.

## Introduction

The discussion around probabilities of occurrence of (perceived) obstetric risks, theories of risk and risk perceptions of women giving birth and of obstetric caregivers, from both a medical and a social science perspective, has been ongoing for decades and remains relevant [1–9]. There is a tension between perceiving (negative) events in peripartum care as fate and the healthcare professionals’ sense of personal responsibility for the pregnant or labouring woman. They act under the belief that these events can be influenced or prevented through interventions [10]. Accordingly, the minimum requirement for engaging with risk, namely the ability to influence the future and avoid events through preventive action [11], indicates that active examination of the subjects of risk and risk perception of obstetric healthcare professionals during peripartum care is called for [10]. Furthermore, while there has been a steady increase in obstetric intervention rates, there are large variations in regional and national implementation [12–16], and various strategies exist at different levels, e.g., systemic or political [17], to address the rising rates of intervention. Another strategy is focussing on decision-making, inviting an examination of factors influencing the decisions made by obstetric healthcare professionals [18–20]. Risk perception is fundamental to decision-making, therefore its exploration is essential to gaining a comprehensive understanding of the decision-making process [21].

Research from other disciplines has already demonstrated that personal or systemic factors can have an impact on healthcare outcomes, e.g., as measured by transfer rates or mortality [22–26]. This connection was identified in the field of obstetrics decades ago under the keywords ‘*physician factor’* [27, 28] or ‘*physician effect’* [29]. Numerous studies have examined the associations between personal or systemic factors, such as gender, years of experience, annual number of births in a hospital per year, hospital size and level of acuity, attitude to risk in obstetric care and implementation of interventions [2, 12, 30–37]. The findings, however, have been inconsistent. While some studies did not find a correlation between the risk perception or risk willingness of obstetric healthcare professionals and selected interventions, others suggested that risk perception, along with other personal or systemic factors, may indeed be linked to decisions regarding implementation of interventions.

Despite the large amount of research, we are not aware of any study specifically examining the association between risk perceptions of obstetric healthcare professionals, personal and systemic factors, and the resultant influence on decision-making or implementation of obstetric interventions. The aim of this explorative study was therefore to answer the research questions (i) Are personal and systemic factors associated with the risk perception of obstetric healthcare professionals? And (ii), Do differing risk perceptions influence the decisions made by these professionals in peripartum care? This study thus contributes to gaining more information on obstetric healthcare workers’ decision-making and influencing factors in order to obtain quantifiable information to address the large variations in intrapartum intervention rates.

## Methods

### Study design and population

This cross-sectional survey using case vignettes is the quantitative second part of a mixed-methods-study [38, 39], and uses an exploratory sequential mixed-methods design [40, 41]. Participants were clinically active midwives and obstetricians who, at the time of the survey, were currently working or had worked in delivery in the past 24 months as an employed/caseload midwife or obstetrician and who had qualified more than 1 year ago. With the aim of generating a non-probabilistic sample from the basic population of midwives and obstetricians working clinically in Germany, a recruitment letter and information flyer were sent to all obstetric clinics in Germany (*N* = 638) which existed at the time of the survey to the knowledge of the author group and professional organisations. Participants were also invited via general calls for participation on social media platforms. Details of the recruitment strategy can be found in the study protocol [38]. Due to the exploratory design of the survey and as the aim was to invite as many midwives and obstetricians working in birth suites/labour wards as possible to participate, no formal sample calculation was carried out. Since there is only data on how many midwives and obstetricians work clinically in Germany, without distinguishing whether they work in birthing or not [42, 43], we estimated the number of professionals working in the clinics invited to participate during the study period at around 10,000.

### Data collection

The survey was conducted electronically over a period of 6 weeks in January and February 2021 on the Unipark platform using the online software EFS-Survey. A direct link to the website was provided within the recruitment letter, information flyer and on social media platforms. Participants were asked to provide demographic data (see Table 1) and to rate on a 6-point Likert scale how risky they thought four obstetric case descriptions ‘were. In addition, they were asked to rate how likely they thought it was that certain interventions would be carried out in such a situation (i.e., involving another professional, performing electronic foetal monitoring, carrying out a vaginal examination, offering analgesia, offering an epidural, administering oxytocin, using alternative methods to promote contractions, administering tocolysis or advising caesarean section). Completion of the survey took about 20 minutes. The case vignettes were constructed using the results of the first part of the mixed methods design (described in detail in [38, 39]), whereby the fourth case has been analysed separately due to its special nature and is not part of this publication. Details on the case vignettes and their respective characteristics are shown in Box 1 in the Supplementary Material 2).

**Table 1:** Characteristics of the participants

Characteristic	N or Mean (SD)	%	Range
Age	42,2 (11,43)		22–66
Professional experience (years)	13,9 (10,58)		1–44
Job title			
Midwife	676	81.7	
Gender	n.a.		
Ob/Gyn/doctor	151	18.3	
Gender			
female	109	75.2	
male	42	27.8	
Occupation^a^			
Employed midwife	453	54.8	
Employed midwife with management function	117	14.1	
Freelance/caseload midwife	23	2.8	
Freelance/caseload midwife on duty	83	10.0	
Junior resident	43	5.2	
Hospital Ob/Gyn	17	2.1	
Hospital senior Ob/Gyn	60	7.3	
Head Ob/Gyn	25	3.0	
Locum Ob/Gyn	6	0.7	
Number of hours worked (per week)			
< 10 h	12	1.5	
10–20 h	124	15.0	
20.5–30 h	217	26.2	
> 30 h	474	57.3	
Annual number of births^b^			
≤ 500	51	6.2	
501–1000	203	24.5	
1001–1500	196	23.7	
1501–2000	167	20.2	
2001–2500	91	11	
> 2500	119	14.4	
Level of care^b, c^			
I (highly specialised obstetric and neonatal care)	315	38.1	
Level II (regional referral hospital)	78	9.4	
III (Perinatal Clinic)	66	8.0	
IV (Hospital)	364	44.0	
Care model^b^			
Midwife-led care	119	14.4	
Standard	708	85.6	

*N* = 827

n.a.: not asked; SD: standard deviation

^a^in each case in the clinical / birthing setting

^b^of the current or last workplace

^c^Explanatory note regarding the levels of obstetric care in the German clinical setting: ‘Level I Regional Perinatal Healthcare Centre’ refers to hospitals with the capacity for providing the highest level of obstetric and neonatal care, i.e., care of premature neonates < 29/40 and/or estimated weight of 1250 g; multiple pregnancies of triplets or greater; severely unwell mothers and/or neonates likely to need surgery (e.g., gastroschisis, heart defects etc.). ‘Level II Perinatal Healthcare Centre’ refers to hospitals with the capacity to care for premature neonates from > 29 + 0–31 + 6 gestation and/or 1250–1499 g estimated weight; severe growth restriction (< 3rd percentile); pregnant women with severe pregnancy-induced illness such as HELLP syndrome or pre- eclampsia. ‘Level III Perinatal Clinic’ refers to hospitals with the capacity to care for premature neonates 32 + 0–35 + 6 gestation and/or at least 1500 g estimated weight; growth restriction between the 3rd and 10th percentiles; pregnant women with insulin-requiring gestational diabetes where the baby was not expected to be born unwell. ‘Level IV hospital’ refers to hospitals able to care for pregnant women and babies from 36 + 0 weeks’ gestation where no complications are expected and none of the criteria listed above are present (Gemeinsamer Bundesausschuss, 2020, translated by author as in Peterwerth et al. 2020)

Certain filter questions led to premature termination of the questionnaire, e.g., if the inclusion criteria were violated. A pretest to check the content and technical aspects of the survey took place shortly before the survey began with people from the target population, academics from the department and those with expertise in questionnaire design (*n* = 33). Based on the results and feedback from the pretest, the survey was adapted accordingly, e.g., wording or layout changes. In addition, midwives were not asked to specify gender identity, as the low number of male midwives in German-speaking countries meant that participant anonymity could not be guaranteed. The questionnaire could only be concluded if all questions were answered fully.

### Statistical analysis

Continuous data were analysed descriptively by calculating measures of location (minimum, maximum, quartiles, means) and measures of dispersion (standard deviation, interquartile range, span width). Categorical and binary data were presented by calculating absolute and relative frequencies within frequency tables.

In order to examine the association between risk perception and personal or systemic factors, the mean value of the risk assessments of cases 1 to 3 was calculated across the various case descriptions. Low values stood for a tendency towards low risk perception, higher values for a tendency towards a higher risk perception. Depending on the scale level of the independent variable, a t-test for independent samples, an ANOVA or a Pearson, respectively Spearman, correlation was calculated. The test for normal distribution was carried out by means of a Q-Q plot, and the verification for outliers was performed through a box plot analysis. In cases where the prerequisites for parametric tests were violated, non-parametric tests (Mann-Whitney-U, Kruskal-Wallis test) were used.

A score for assessing readiness for intervention was formed to investigate the influence of risk perception on decision-making related to performing interventions, based on the rating of the 3 case vignettes on the 6-point Likert scale. The variables ‘perform electronic foetal monitoring’, ‘offer analgesia’, ‘offer epidural’, ‘administer oxytocin’ and ‘advise caesarean section’ were taken into account (Min 0, Max 70; see Table 2). A multiple linear regression was performed with the dependent variable ‘Intervention Readiness Score’ and the independent variables for which the bivariate analysis showed a significant difference. While the formal statistical prerequisites of uncorrelated residuals and independence of the residuals in addition to no multicollinearity were fulfilled (Durbin-Watson value close to 2 or VIF values less than 5 as well as condition index less than 30), the examination of the residuals by means of residual statistics and P-P diagram showed that there were no normally distributed residuals. Bootstrapping was applied due to the violated requirement of normally distributed residuals. The results of the multiple linear regression are presented with the regression coefficient and confidence intervals.

**Table 2:** Composition of the risk assessment and intervention readiness score

Score	Evaluation			Values	Interpretation
Risk Perception	Risk assessment cases 1 to 3 *6-point Likert scale*			Min 3 Max 18	low values = tendency towards lower risk perception; higher values = tendency towards higher risk perception
Intervention readiness	Assessment of the probability of carrying out selected interventions for 3 cases Interventions to choose from (*6-point Likert scale*):			Min 0 Max 70	low values = tendency to be less willing to intervene; higher values = tendency to be more willing to intervene
		Cases 1 & 2			
			perform electronic foetal monitoring,		
			offer analgesia,		
			offer epidural,		
			administer oxytocin,		
			advise caesarean section		
		Case 3			
			offer analgesia		
			offer epidural		
			administer oxytocin		
			advise caesarean section		

The significance level was set at α = 0.05. To adjust for multiple testing, an α-adjustment was performed using Bonferroni correction to neutralise α-error accumulation. In addition to the p-values, the effect size according to Cohen, to indicate the strength of the tests, and Nagelkerke’s R^2^, to assess model quality, are reported for significant results. The statistical analysis was carried out using SPSS Statistics 29.0 (IBM Inc.).

## Results

### Sample

During the survey period, the survey page was accessed 1,312 times and the participation button clicked 1,289 times, with 23 people (1.8%) refusing consent and thus participation without giving a reason. 864 participants completed the online questionnaire, which corresponds to a completion rate of 67%. 37 participants (4.3%) triggered a filter question that led to an early termination of the survey. In the end, a total sample of 827 was generated. The participants were aged between 22 and 66 years (M = 42.2; SD 11.34). 676 participants (81.7%) gave their professional title as midwife and 151 participants (18.3%) as (specialist) doctor for obstetrics and gynaecology. The sample is representative of the population of midwives with regard to the characteristics of gender, age, professional activity, occupation, number of hours worked and level of acuity of the current workplace. With regard to the group of participating doctors, the sample is only representative for the characteristic gender, but not for age and occupation. See Table 1 for further details on participants’ characteristics.

### Results of the association of risk perception and personal and systemic factors

The results answering the first research question, whether person-related and system-immanent factors experienced by obstetric healthcare professionals are related to their risk perception during the care of women in labour, are presented below.

There were a total of 827 valid responses available for the assessment of risk perception for the three case descriptions. Low values stood for a tendency towards low risk assessment, higher values for a tendency towards higher risk assessment, where of possible scores, 3 was the minimum and 18 the maximum (see Table 2). The mean value of the ratings was 7.8 (SD 1.55). The lowest value was 4 (*n* = 2), the highest value 14 (*n* = 1). Significant correlations were found for both personal and systemic factors.

#### Investigating the association of risk perception and personal factors

The mean risk assessment score (RAS) of female participants was 7.8 (SD 1.53; min. 4.00, max. 14.00) and that of male participants was 8.6 (SD 1.74; min. 6.00, max. 14.00). This represents a statistically significant correlation between the risk perception of female compared to male participants, with female participants’ RAS 0.8 points lower on average (95% CI [-0.31; -1.23]), *p* = .001, *d* ≈ 0.2; see Table 3). It should be noted that the majority of participants were midwives, and therefore assigned the gender female (see discussion). No significant relationship was found on comparison of the mean values of doctors by gender alone (95%-CI [-0.74; 0.39]), *p* = .535), however comparison of the mean values of midwives and female doctors showed a statistically significant relationship (95%-CI [-1.01; -0.40], p = < .001). As expected, there was a statistically significant difference between risk perception and professional qualification, with midwives scoring on average 0.8 RAS points lower than doctors (95%-CI [-0.673; -0.317]), *p* < .001, *d* ≈ 0.2). The mean RAS of midwives was 7.7 (SD 1.51) and that of doctors was 8.4 (SD 1.56).

**Table 3:** Results of the t-tests of the association between risk perception and personal / systemic factors

Parameter	N	M	SD	t	P	Cohen’s d
Gender						
Female	785	7.8	1.53	825	< 0.001	*0.2*
Male	42	8.6	1.74			
Job title						
Midwife	676	7.7	1.51	825	< 0.001	*0.2*
Doctor / Consultant	151	8.4	1.56			
Care model^a^						
Midwife-led care	116	7.61	1.50	821	< 0.065	
Standard	707	7.83	1.56			

M: Mean, SD: Standard Derivation

^a^of the current workplace; *N* = 823

A significant correlation for the personal factor *occupation* was also found (*p* < .001; see Table 4*).* Subsequent post-hoc tests (Bonferroni) showed that the only significant differences were between employed midwives vs. senior Ob/Gyn consultants^1^ (*p* < .001, *r* ≈ .2), and employed midwives vs. Ob/Gyn consultants (*p* = .012; *r* ≈ .2). Here, the mean RAS of employed midwives was 7.62 (SD 1.53), that of Ob/Gyn consultants 8.76 (SD 0.83), and that of senior Ob/Gyn consultants 8.50 (SD 1.47). Here, too, midwives tended to have a lower risk perception overall, as measured by the risk assessment score across the case descriptions.

**Table 4:** Results of Mann-Whitney-U or Kruskal-Wallis tests

Parameter	M*	SD	df	Chi-Square	p	Effect size
**Professional occupation**			*8*	*38.201*	*p* < .001	
Employed midwife	7.6	1.53			*p* < .001	0.2
Ob/Gyn consultant	8.8	0.83				
Senior Ob/Gyn consultant	8.5	1.47			*p* = .012	0.2
**Numbers of hours worked (per week)**			*3*	14.527	*p* = .002	
< 10 h	7.92	1.24				
10–20 h	7.62	1.68				
20.5–30 h	7.55	1.37			*p* = .005	0.1
> 30 h	7.96	1.58				
**Annual Number of births** ^**a**^			7	827	*p* = .132	
≤ 500	7.80	1.56				
501–1000	7.76	1.55				
1001–1500	7.65	1.55				
1501–2000	7.66	1.44				
2001–2500	7.99	1.65				
> 2500	8.18	1.55				
**Level of care** ^**a**^			3	823	*p* = .023	
Level I (specialist obstetric and neonatal care)	7.97	1.58			*p* = .016	0.1
II (regional referral hospital)	7.86	1.59				
III (Perinatal Clinic)	7.68	1.52				
IV (hospital)	7.63	1.49				

*Mean values of the risk assessment scores

^a^at the current workplace

Regarding years of professional experience, although there was a statistically significant positive correlation between these and the summarised risk perception, this was very weak (correlation coefficient *r* = .074; *p* = .033; see Table 5). Moreover, a categorical division of years in the profession (1, 2–3, 4–5, 6–15 and 16–25 years) showed no significant correlation (*p* = .372).

**Table 5:** Results of the correlations of the association between risk perception and personal and systemic factors

Variable	M	SD	1	2	3
1. Risk assessment	7.8	1,55	-		
2. Years of professional	13.9	10,58	0.074*	-	
Experience					
3. Annual Number of births at the current workplace	1593	870,10	0.076*	-	-

*N* = 827

^*^p < .05

The mean RAS of participants working part time < 10 h/week was 7.92 (SD 1.24), those working 10–20 h 7.62 (SD 1.68), those working 20,5–30 h 7.55 (SD 1.37) and those working ≥ 30,5 h 7.96 (SD 1.58). Here too, a statistically significant relationship was found between risk perception and number of hours worked (*p* = .002; see Table 4*)*. Subsequent post-hoc tests (Bonferroni) showed that only the risk perception of those participants working part-time 20,5–30 h/week differed statistically significantly from those of participants working ≥ 30,5 h (*p* = .005, *r* ≈ .1), with the latter group having a statistically significantly higher risk perception.

#### Investigating the relationship between risk perception and systemic factors

A statistically significant positive correlation was found regarding the annual number of births in the obstetrician’s current workplace and the risk assessment summarised, but this was also a very weak correlation (correlation coefficient *r* = .076; 95% CI [0.006; 0.145]; *p* = .029; see Table 5). Moreover, when the annual number of births was divided into 8 categories (in steps of 500), no significant difference was found (*p* = .132; see Table 3*)*.

There was a significant difference between the combined risk assessment and the level of care at the current workplace (*p* = .023; see Table 4*).* In subsequent post-hoc tests (Bonferroni) it was found that only the results of those participants working in a Level IV^2^ hospital differed significantly from those working in a Level I hospital (*p* = .016, *r* ≈ .1), with the mean value of the former being 7.63 (SD 1.49), whereby the latter had a mean of 7.97 (SD 1.58).

RAS was found to be on average 0.284 points lower for participants working in labour wards with the option of midwife-led birthing care. There was no statistically significant difference between the risk perception of participants working in a labour ward with, vs. without the option of midwife-led birthing care (95%-CI [-0.018; -0.587], *p* = .065).

The results of the survey give rise to the hypothesis that personal factors and those immanent to the system such as *professional qualification, occupation, number of hours worked* and *level of care of the current workplace* are associated with the risk perception of obstetric healthcare professionals. Overall, however, the correlations were weak.

### Results of the association between risk perception and implementation of interventions

The results answering the second research question, whether different risk perceptions could influence obstetric healthcare professionals’ decision-making in the care of women in labour, are presented below.

A total of 824 valid responses were available for the calculation of an intervention readiness score of the three case descriptions on a 6-point Likert scale. Bivariate analysis showed a significant difference for the variables *gender, occupation, professional experience (in years), number of hours worked* and *risk perception*, which is why these were incorporated in the regression model using the inclusion method.

The F-test shows that the regression model as a whole makes a significant explanatory contribution (F(5,818) = 49.094; *p* < .001). With the exception of gender, all predictors (risk perception, occupation, years of professional experience and number of hours worked) were significant in this model (see Table 6). The BCa confidence intervals did not include the value zero for all significant predictors, so this result was robust. If the risk score increases by 1 unit, the intervention readiness score increases by 1.86 (95% CI: [1.511; 2.201]). A medical profession causes the intervention readiness score to be 4.09 points (95%- CI: [2.409; 5.731]) higher compared to ‘midwife’, regardless of all other factors. For each year of professional experience the intervention readiness score increases by 0.055 (95% CI: [0.007; 2.201]). Working more than 30 hours per week) results in the intervention readiness score being 1.66 points higher (95%- CI: [0.600; 2.697]), regardless of all other factors. The survey showed that risk perception, occupation, number of years of professional experience and number of hours worked have a significant influence on obstetric healthcare professionals’ readiness to intervene. Overall, the model explained 23% of the variance in the intervention readiness score, which, according to Cohen 1988 [44], is a weak effect.

**Table 6:** Results of the multiple linear regression

		Dependent variable: Intervention readiness score						
Coefficients		*b*	SE	*β*	*t*	95% CI		*p*
						*LL*	*UL*	
(constant)		-7.443	2.074		-3.846	-11.572	-3.278	< 0.001
Risk assessment		1.864	0.176	0.354	11.271	1.511	2.201	< 0.001
Gender (female)		0.506	1.676	0.014	0.378	-2.785	3.858	0.706
Medical profession as Ob/Gyn/doctor		4.090	0.839	0.194	5.332	2.409	5.731	< 0.001
Years in profession		0.055	0.025	0.072	2.269	0.007	0.104	0.024
Hours worked > 30/week		1.656	0.539	0.101	3.167	0.600	2.697	0.002

*Note N* = 823; corr. R² = 0.226; F(5,818) = 49.094; *p* < .001

CI = confidence interval; *LL* = lower limit; *UL* = upper limit

^a^ Confidence intervals and standard errors per BCa bootstrapping with 5000 BCa samples

## Discussion

### Main findings

The results of the survey showed that personal and systemic factors are associated with the risk perception of obstetric healthcare professionals. Midwives tended to have a lower risk perception than obstetricians, whereby a differentiation by occupation showed that employed midwives in particular tended to have a lower risk perception than Ob/Gyn consultants. Participants working between 20.5 and 30 h/week also showed a lower risk perception than those working > 30 h/week. Participants working in a Level I hospital (highly-specialised obstetric and neonatal care) tended to have a slightly higher risk perception compared to participants from a Level IV hospital. Based on the results, we also assume that risk perception, profession, years of experience and the proportion of hours worked by obstetric healthcare professionals can be used to explain variations in decisions made regarding obstetric interventions, with a medical profession (doctor) being the most important factor by a large margin, followed by risk assessment. However, the differences we found between groups were small and interpretations should be made cautiously.

Particularly interesting is the result that a significant difference between the professional qualification or occupation could be determined both in the assessment of risk and the subsequent assessment regarding the implementation of interventions. While the difference in risk perception, as assessed by the summary risk assessment score, was rather small, the occupation of “doctor” compared to “midwife” led to a significant increase in the willingness to intervene, even if it was a weak effect overall. The aim of this research was not to evaluate whether the delivery of interventions within the case vignettes was ‘correct’ or not. Rather, identification of these differing viewpoints highlights the potential for interdisciplinary conflict in the care of women in labour, which could have a negative impact on that care. The medicalisation of birth and its influence on the risk perception of obstetric healthcare professionals is a frequently discussed topic [3, 45] and was one of the motivating factors for this research project. The description of and distinction between the social and medical models are regularly discussed and addressed in the literature, with a general tendency to attribute the social model to midwives and the medical model to medical professionals [1, 46]. It is not possible to determine whether this could be an explanation for the differences in the intervention readiness score, but would be an exciting topic for further research. Regardless, these findings confirm the necessity of developing strategies to improve interdisciplinary relationships, as advocated by other researchers in the field [47]. It is important to foster a shared understanding between professional groups about the need to refrain from, or the real need for the implementation of interventions.

Another surprising result is that the willingness to intervene increased with years of professional experience. We cannot explain with certainty why this was the case in this survey, but it does confirm the results of other research. According to the systematic review by Panda et al. 2018 [31], clinicians’ decision-making was influenced by their level of experience, with obstetricians of greater seniority and experience showed an increased risk of performing or approving a CS. Fear of legal consequences was identified as an influencing factor for interventions, respectively CS. Fear of legal consequences and the influence on one’s own practice were also identified in the first (qualitative) part of this mixed-methods design [39], and we assume that with increasing professional experience the awareness of legal consequences increases. The use of availability heuristics and thus possibly overestimation of risks was also evident in the qualitative study. Both could be a possible explanation for the increase in willingness to intervene with increasing professional experience.

It should be noted that we are currently not yet in a position to interpret the statistically significant difference in terms of its clinical extent. Further research on this topic would be desirable.

### Interpretation (in light of other evidence)

The results of this study build upon various other research findings. Nippita et al. 2017 found that variations in the decision-making process, in this case regarding induction of labour, were influenced by the obstetrician’s perception of medical risk during pregnancy, which in turn was influenced by their personality and knowledge [12]. Similarly, Healy et al. 2017 describe how risk perception affects the implementation of care practices for normal birth and low-risk women giving birth in general [3].

In another study, Tracy et al. 2006 [32] investigated the relationship between the number of births/year in a hospital and a negative birth outcome, measured by various interventions such as induction of labour, epidural anaesthesia or unplanned caesarean, and whether hospital size was an independent risk factor for their occurrence. The author group disagrees with the assumption that a low number of births leads to a worse outcome. In our study, although there was a statistically significant association between the risk score and the number of births at the obstetrician’s current workplace, this was only very small and accordingly there was only a statistically very weak effect, so that our results clinically support the conclusion of Tracy et al. 2006. The assessment according to the level of care of the current workplace is somewhat different. Here, our study showed a significant difference in risk assessment between participants working in a Level IV hospital compared to those in Level I hospitals (high acuity). While Tracy et al. 2006 did not differentiate according to the level of care, there are other research studies that make a comparable classification of units, including Mead and Kornbrot 2004 and Wiklund et al. 2012 [2, 33]. While Mead and Kornbrot 2004 [2] found that midwives who worked in units with a high rate of intrapartum intervention generally had a higher perception of risk than those who worked in a unit with a lower rate of intervention, Wiklund et al. 2012 [33] came to a slightly different conclusion. The authors showed that midwives working in low-risk units had a significantly different attitude towards interventions, namely more reserved, than their colleagues in standard care units. However, they found no difference in risk perception of the groups regarding three vignettes, and describe these results as partially conflicting with those of Mead and Kornbrot 2004. In our study, participants from Level IV hospitals had the lowest mean risk assessment score, while those from Level I hospitals had the highest. In relation to the readiness to intervene score, however, this pattern was not repeated, which is why in summarising our study we can say that the level of care of the workplace was related to the assessment of risk perception, but did not seem to have any influence on decision-making as measured by readiness to intervene. Thus, these results are again partly contrary to Wiklund et al. 2012 [33]. In relation to our study, however, it should be noted that participants who worked in Level I and Level IV hospitals were particularly strongly represented. It would be interesting to investigate whether this distribution is a reflection of the number of individual hospitals with corresponding levels of acuity, or an indication that participants from the corresponding hospitals are generally more interested in and motivated to examine this topic. We cannot make any statement about this at present and further research in this regard would be desirable.

### Strengths and limitations

#### Strengths

This study is the first to specifically investigate the association between the risk perception of obstetric healthcare professionals and personal and systemic factors and to link it to decision-making. The findings can help to explain the sometimes significant regional and national variations in obstetric intervention rates, e.g. varying caesarean section (15-37%) or episiotomy rates (5–70%) [12–16].

Although there was no formal power calculation due to the pre-planned recruitment strategy, the sample size is a strength of this survey. The study included a large clinically relevant sample and the number of participants exceeded the expectations of the research group, reflecting a high level of interest of healthcare professionals in the topic. Studies on both decision-making and risk perception are otherwise often characterised by smaller samples [33, 35].

In addition, this study uses a mixed-methods approach and links the qualitative research findings of the first part [39] with the second, quantitative one. The dovetailing of the results with the very large sample size, even taking the limitations into account, nevertheless strengthens the conclusions drawn about the overall population.

Furthermore, the inclusion of the two professional groups primarily involved in clinical obstetric care, namely midwives and doctors/obstetricians/gynaecologists, is also a strength, as the professions are often considered in isolation [30, 33, 48, 49], despite the fact that they almost always work together clinically.

In addition to the results presented here, the data could be further analysed to gain further insights, e.g., exploring the extent to which years of experience and the current workplace are connected.

### Limitations

The use of hypothetical case descriptions cannot fully replicate real clinical situations, as clinical decisions are based on verbal, visual and intuitive information available to clinicians, as well as the individual’s manner of interpretation and action [1]. This restricts transferability of the results, limiting the validity of the study. However, like other research groups, we see a clear advantage in the use of case vignettes as they provide the respondents with uniform information, counteracting the influence of other contextual factors which are not the subject of the study, and permitting a focus on the research area [35, 50]. The creation of the case descriptions as a result of group discussions in the first part of this research project [39] enhances their validity, an approach which has already been shown to be practical in other research studies [35]. We chose not to conduct participatory observations in the field, as they had been rejected for ethical reasons by the ethics committees of previous studies on the influence of risk perception [3]. Nevertheless, it should be mentioned that the unclear extent of concurrence between hypothetical and actual behaviour and thus the assessment of the prognostic and external validity of the vignette method must be taken into account when interpreting the results [51]. In addition, we are not able to compare or to check if the interventions presented would or would actually not have been carried out, or in what way this is linked to maternal and foetal outcomes. It would be desirable for such studies to be carried out as a continuation of this research. Likewise, due to the chosen method, interpretation regarding a cause-effect principle is not possible. This is not strictly a limitation, however, as this research project was explorative and hypothesis-generating in nature.

Furthermore, these exemplary case vignettes represent only a small section of the spectrum of intrapartum care which is not representative of other risky situations or complications that may occur during intrapartum care; thus the results should not be generalised to represent obstetric risk assessment on the whole.

There were clear ceiling effects for certain options, in the assessment of both risk perception and the interventional readiness score. Although this was partly intended or foreseeable in terms of content (e.g., for case vignette 2, as this corresponded to a description of a physiological birth process without special features or risk factors), it may have influenced the results. Nevertheless, we deem this positive overall, because we see it as an indication of a roughly similar understanding of care, which is to be assessed positively per se.

Due to the low number of male participants (*n* = 42), the evaluation of the influence of gender on risk perception and willingness to intervene is therefore biased. Further, as there were only a very small number of male midwives in Germany at the time of the survey, to preserve anonymity those participants who gave their profession as midwives were not asked about their gender identity. This was a consequence of feedback from the pretest. A more diverse group of participants would be desirable for further research on this topic.

In order to comply with the data protection regulations for anonymous participation, no IP addresses or cookies were stored. This means that there is a theoretical possibility that the questionnaire was filled out more than once by one person. However, we consider this possibility to be low, since studies show that this rarely occurs or is unlikely to bias the results [52].

The study period was in 2021 and therefore during the Covid-19 pandemic period. We do not believe that the pandemic period may have influenced participants’ interpretation of the vignettes, as our aim was not to survey the general perception of risk, but to focus on its association with personal and systemic factors and their influence on decisions using specific descriptions of cases. Nevertheless, it is possible that the general perception of risk among professionals during the pandemic and post-pandemic period may have had influenced our survey, which must be taken into account when interpreting the results.

## Conclusion

Despite the explorative and hypothesis-generating character of this research project, it provides important insights into the risk perception and decision-making of obstetric healthcare professionals. For the first time, insights were gained into whether and to what extent personal factors of obstetric healthcare professionals or systemic factors of the work setting are associated with their risk perception. The results of this study lead to the hypothesis that the obstetric healthcare professionals’ qualification, occupation, number of hours worked and the level of care of the workplace are related to their risk perception. In turn, risk perception itself made a significant contribution to explaining differences in willingness to intervene, which is why risk perception appears to have an influence on obstetric healthcare professionals’ decision-making. Other significant variables in this context were occupation (obstetrician/gynaecologist vs. midwife) and, to a much lesser extent, years in profession and number of hours worked. It should be noted that these were very weak effects overall.

The significant variations in intervention rates need to be addressed in terms of providing the highest quality and best possible care for childbearing women and their families. To this end, developing strategies to improve interdisciplinary relationships and collaboration is of great importance. Whether and to what extent a woman in labour experiences obstetric interventions should not depend on the obstetric healthcare professional’s perception of risk but should in the best case be influenced by an appropriate response to the individual birth situation based on current scientific knowledge. The aim should always be to provide the best possible care, taking into account the individual situation of the woman giving birth, her wishes and the current evidence base.

### Electronic supplementary material

Below is the link to the electronic supplementary material.

**Supplementary Material 1:** Detailed description of the different medical professions

**Supplementary Material 2:** Infobox case vignettes

## Data Availability

The data supporting the conclusions of this article are included in the article or additional files. Additional datasets can be requested from the main author (NP).

## Footnotes

1 For a detailed description of the different medical professions see Supplementary Material 1.

2 A description of the levels of care in the German clinical setting can be found in the footnotes of Table 1.
