# Supplementary material for: Association of personal and systemic factors on intrapartum risk perception and obstetric intervention rates: a cross-sectional study
Source: BMC Pregnancy Childbirth. 2024 Feb 22;24:155. doi: 10.1186/s12884-024-06338-w (PMC10882933; doi:10.1186/s12884-024-06338-w)
Supplement: Supplementary file 1 — Supplementary Material 1: Detailed description of the different medical professions [file 12884_2024_6338_MOESM1_ESM.docx]

**Additional file 1: Detailed description of the different medical professions**

**Junior resident:**

- equivalent to medical resident/ junior medical officer (JMO)
- has completed medical training and holds a medical license
- responsible for direct patient care and undergoing vocational training in a certain specialty
- working under the guidance of more experienced physicians, usually by hospital (senior) Ob/Gyn (consultant/specialist/medical fellow)

**Hospital Ob/Gyn:**

- Equivalent to medical fellow/ consultant/ staff specialist/ specialist medical practitioner
- has successfully completed training in medical specialty (i.e. gynaecology and obstetrics)
- more experienced than a resident doctor
- may undergo training in a sub-specialty
- may make unsupervised medical decisions, has limited medical authority
- may take on leadership roles in patient care and education

**Hospital senior Ob/Gyn:**

- Equivalent to attending physician/ senior consultant/ senior specialist
- must have completed training in medical specialty and usually also medical sub-specialty
- responsible for all aspects of patient care in his or her ward/team
- supervises and teaches residents and medical students
- may make unsupervised medical decisions, however, may seek advice from medical director/Chief

**Head Ob/Gyn**

- Equivalent to medical director/ chief attending physician/ head consultant
- Is usually head of department, holds the most power and responsibility in a hospital or clinic
- oversees all medical staff in department
- makes ultimate medical decisions, may veto in decisions made by all staff in team
- usually enforces hospital policies, develops treatment guidelines, coordinates research activities, and monitors the quality of patient care

**Locum Ob/Gyn:**

- Holds position as external hospital physician/ affiliated physician/ locum/ visiting medical officer (VMO)
- is not employed by the hospital, i.e. no staff
- responsive for medical care / medical procedures of certain inpatients as external person
- may make unsupervised medical decisions
