# Supplementary material for: Association of personal and systemic factors on intrapartum risk perception and obstetric intervention rates: a cross-sectional study
Source: BMC Pregnancy Childbirth. 2024 Feb 22;24:155. doi: 10.1186/s12884-024-06338-w (PMC10882933; doi:10.1186/s12884-024-06338-w)
Supplement: Supplementary file 2 — Supplementary Material 2: Infobox case vignettes [file 12884_2024_6338_MOESM2_ESM.docx]

**Additional file 2: Infobox case vignettes**

The specifics of the different cases are described below:

**Case 1:** 26 y. G2P1, 39+2 weeks, history of Caesarean for breech presentation, nil other significant medical history; regular contractions, cervix 4 cm dilated, presenting part (head) engaged; membranes intact; unremarkable CTG 🡪 i.e., situation with a (supposed) obstetric risk factor because of the previous caesarean, but otherwise describes a physiological birth process with no other special features.

**Case 2:** 29 y. G1P0, 40+0 weeks, nil other significant medical history; in established labour: cervix 6 cm dilated, presenting part (head) engaged; membranes intact; unremarkable CTG🡪 i.e.,a physiological birth situation without any further specificities or risk factors. The details were almost the same as in Case 1 apart from being a nullipara, so no previous CS.

**Case 3:** 32 y.o.G2P1, 36+5 weeks; previous preterm birth; gestational diabetes, diet-controlled; premature rupture of membranes 18 hours ago, irregular contractions; cervix dilated 4 cm, presenting part (head) engaged; discharge of small amount of clear amniotic fluid; pathological CTG- baseline 165 spm, oscillations > 5 bpm and < 25 bpm, no decelerations; irregular contractions every 5 to 10 min. 🡪 was intended to describe a birth situation which may be assessed differently within the different clinic care levels. The medical risk factors here were expected preterm birth, diet-controlled gestational diabetes and premature rupture of membranes with rising inflammatory markers and a pathological CTG.
